# Downregulation of lncRNA FGF12-AS2 suppresses the tumorigenesis of NSCLC via sponging miR-188-3p

**DOI:** 10.1515/med-2020-0219

**Published:** 2020-10-08

**Authors:** Lili Zhou, Chen Xing, Dongxia Zhou, Rong Yang, Maohuai Cai

**Affiliations:** Department of Oncology, Yancheng Second People’s Hospital, No. 135 Kaifang Avenue, Yancheng 224003, Jiangsu, China

**Keywords:** NSCLC, FGF12-AS2, miR-188-3p, NCAPG2

## Abstract

**Background:**

Non-small-cell lung carcinoma (NSCLC) seriously threatens the health of human beings. Aberrant expression of lncRNAs has been confirmed to be related with the progression of multiple malignant tumors, including NSCLC. LncRNA FGF12-AS2 has been considered to be upregulated in NSCLC. However, the mechanism by which FGF12-AS2 promotes the tumorigenesis of NSCLC remains elusive.

**Methods:**

Gene and protein expressions in NSCLC cells were measured by q-PCR and western blot, respectively. CCK-8 and immunofluorescence staining were performed to detect the cell proliferation. Cell apoptosis was tested by flow cytometry. Transwell assay was used to detect the cell migration and invasion. Finally, the dual luciferase report assay was used to verify the relation among FGF12-AS2, miR-188-3p, and NCAPG2.

**Results:**

Downregulation of FGF12-AS2 significantly inhibited the proliferation of NSCLC cells via inducing apoptosis. In addition, FGF12-AS2 silencing notably suppressed the migration and invasion of A549 cells. Meanwhile, FGF12-AS2 modulated the progression of NSCLC via regulation of miR-188-3p/NCAPG2 axis. Finally, knockdown of FGF12-AS2 inhibited the tumorigenesis of NSCLC via suppressing the EMT process of NSCLC.

**Conclusion:**

Downregulation of lncRNA FGF12-AS2 suppressed the tumorigenesis of NSCLC via sponging miR-188-3p. Thus, FGF12-AS2 may serve as a potential target for the treatment of NSCLC.

## 1 Introduction

Lung cancer is one of the most frequent and lethal malignancies. In addition, non–small-cell lung carcinoma (NSCLC) accounts for about 80% of the lung cancer-related morality [1,2]. Although chemotherapy and molecular-targeted therapy have been improved recently, the prognosis of NSCLC remains poor. The recurrence and metastasis of cancer cells are critical difficulties affecting the clinical management of NSCLC [3]. Hence, it is urgent and necessary to find new strategies that could suppress the invasiveness and metastasis of NSCLC cells.

Long noncoding RNAs (lncRNAs) are a class of noncoding RNA transcripts that are more than 200 bp long [4]. LncRNAs are key mediators that are notably participated in the progression of multiple diseases [5,6]. Recent studies have shown a close correlation between lncRNAs and cancer progression [7,8]. For instance, knockdown of lncRNA XIST could increase the chemosensitivity of NSCLC cells via downregulation of autophagy [4]. Meanwhile, lncRNA FGF12-AS2 has been found to be upregulated in NSCLC [9]. However, the underlying mechanism remains unclear.

Previous studies have reported that epithelial-to-mesenchymal transition (EMT) leads to early-stage dissemination of tumor cells and promotes the invasion and metastasis of cancer cells [10,11]. EMT is closely related with phenotypic conversion of epithelial cells to mesenchymal-like cells in cell culture conditions [12]. In addition, Tong et al. indicated that EMT process played a key role during the metastasis of lung cancer [13]. On the basis of these backgrounds, we sought to explore the effect of FGF12-AS2 on the process of EMT in NSCLC.

In this study, we aimed to investigate the underlying mechanism of FGF12-AS2 in NSCLCs. Silencing of FGF12-AS2 was confirmed to suppress the NSCLC growth by sponging miR-188-3p and inhibiting the EMT process.

## 2 Material and methods

### 2.1 Cell culture

The lung cancer cell lines (A549 and NCI-H23) and 293T cell line were obtained from the Institute of Biochemistry and Cell Biology of the Chinese Academy of Sciences (Shanghai, China). All cell lines were cultured in RPMI-1640 medium (Gibco, Carlsbad, CA, USA) supplemented with 10% fetal bovine serum (Gibco) and penicillin (100 U/mL). In addition, cells were cultured at 37°C in the presence of 5% CO_2_.

### 2.2 Quantitative real-time polymerase chain reaction

Total RNA was extracted from NSCLC cell lines using TRIzol reagent (TaKaRa, Tokyo, Japan) according to the manufacturer’s protocol. cDNA was synthesized using the reverse transcription kit (TaKaRa, Ver.3.0) according to the manufacturer’s protocol. Real-time qPCRs were performed in triplicate under the following protocol: 10 min at 95°C, followed by 35 cycles of 15 s at 95°C and 1 min at 60°C. The primers for lncRNA FGF12-AS2, miR-188-3p, NCAPG2, β-actin, and U6 were obtained from GenePharma (Shanghai, China). lncRNA FGF12-AS2: forward, 5′-TGAGAAGTCAGGTGTGCGTA-3′; reverse, 5′-AGAGACTTCTTCCAGGCAAC-3′. MiR-188-3p: forward, 5′-TGCGCTCAGCAAACATTTATTG-3′; reverse, 5′-CCAGTGCAGGGTCCGAGGTATT-3′. NCAPG2: forward, 5′-CTGGGGAACTGGCATTTGAC-3′; reverse, 5′-GCTACCCTCACTTTCTCCGA-3′. β-actin: forward, 5′-AGCGAGCATCCCCCAAAGTT-3′; reverse, 5′-GGGCACGAAGGCTCATCATT-3′. U6: forward, 5′-CGCTTCGGCAGCACATATAC-3′; reverse, 5′-AAATATGGAACGCTTCACGA-3′. The relative fold changes were calculated using the 2^−ΔΔCt^ method by the following formula: 2 − (sample Δ*Ct* – control Δ*Ct*), where Δ*Ct* is the difference between the amplification fluorescent thresholds of the gene of interest and the internal reference gene (U6 or β-actin) used for normalization.

### 2.3 Cell transfection

Lentiviral expressing short-hairpin RNA (shRNA1 or shRNA2) directed target FGF12-AS2 and one nontargeting sequence (negative control [NC]) were obtained from Hanbio Biotechnology Co., Ltd (Shanghai, China). Next, FGF12-AS2 shRNA1 or shRNA2 was packaged into lentiviruses. Then, the lentiviral vector DNAs were then transfected into 293T cells including lenti-FGF12-AS2 shRNAs and negative control (NC). After transfection, the cells were incubated at 37°C, and then, the supernatant was collected. Then, supernatants of two FGF12-AS2 shRNAs and NC were filtered into particles. Finally, all NSCLC cells were infected with lentiviral particles according to the manufactures’ protocol. After 48 h of incubation, stable NSCLC cells were then selected by puromycin (2.5 µg/mL, Sigma Aldrich, St. Louis, MO, USA). qRT-PCR assay was used to verify the efficiency of transduction.

For miR-188-3p transfection, A549 cells were transfected with miR-188-3p agonist, miR-188-3p antagonist, or NC by Lipofectamine 2000 according to the previous study [14]. MiR-188-3p agonist, miR-188-3p antagonist, and NC RNAs were bought from GenePharma (Shanghai, China).

### 2.4 CCK-8 assay

A549 or NCI-H23 cells were seeded in 96-well plates (5 × 10^3^ per well) overnight. Then, cells were treated with NC or FGF12-AS2 shRNA2 for 0, 24, 48, and 72 h. 10 µL CCK-8 reagents were added to each well, and cells were incubated for 2 h at 37°C. Finally, the absorbance of NSCLC cells was measured at 450 nm using a microplate reader (Thermo Fisher Scientific).

### 2.5 Cell apoptosis analysis

A549 cells were trypsinized, washed with phosphate-buffered saline, and resuspended in Annexin V Binding Buffer. Then, cells were stained with 5 µL FITC and 5 µL propidium (PI) for 15 min. After that, cells were analyzed using the flow cytometer (BD, Franklin Lake, NJ, USA) to test the cell apoptosis rate.

### 2.6 Dual luciferase reporter assay

The partial sequences of FGF12-AS2 and 3′-UTR of NCAPG2 containing the putative binding sites of miR-188-3p were synthetized and obtained from Sangon Biotech (Shanghai, China), which were then cloned into the pmirGLO Dual-Luciferase miRNA Target Expression Vectors (Promega, Madison, WI, USA) to construct wild-type or mutate type reporter vectors FGF12-AS2 (WT/MT) and NCAPG2 (WT/MT), respectively. The FGF12-AS2 (WT/MT) or NCAPG2 (WT/MT) was transfected into cells together with control, vector-control (NC), or miR-188-3p agonist using Lipofectamine 2000 (Thermo Fisher Scientific) according to the manufacturer’s instructions. The relative luciferase activity was analyzed by the Dual-Glo Luciferase Assay System (Promega).

### 2.7 Fluorescence *in situ* hybridization (FISH) detection

To explore the relation between FGF12-AS2 and miR-188-3p, colocalization of miR-188-3p and FGF12-AS2 on cytoplasm was investigated by FISH detection according to the previous study [15].

### 2.8 Western blot detection

Total protein was isolated from cell lysates by using RIPA buffer and quantified by the BCA protein assay kit (Beyotime, Shanghai, China). Proteins were resolved on 10% SDS-PAGE and then transferred to PVDF (Bio-Rad) membranes. After blocking, the membranes were incubated with primary antibodies at 4°C overnight. Then, the membranes were incubated with secondary anti-rabbit antibody (Abcam; 1:5,000) at room temperature for 1 h. Membranes were scanned by using an Odyssey Imaging System and analyzed with Odyssey v2.0 software (LICOR Biosciences, Lincoln, NE, USA). The primary antibodies used in this study are as follows: anti-NCAPG2 (Abcam, Cambridge, MA, USA; 1:1,000), anti-Bax (Abcam; 1:1,000), anti-XIAP (Abcam; 1:1,000), anti-activated caspase 9 (Abcam; 1:1,000), anti-E-cadherin (Abcam; 1:1,000), anti-N-cadherin (Abcam; 1:1,000), anti-Vimentin (Abcam; 1:1,000), and anti-β-actin (Abcam; 1:1,000). β-actin was used as an internal control.

### 2.9 Immunofluorescence

NSCLC cells were seeded in 24-well plates overnight. Then, cells were treated with NC or FGF12-AS2 shRNA2 for 72 h. Next, cells were blocked with 10% goat serum for 30 min at room temperature and then incubated with anti-Ki67 antibody (Abcam; 1:1,000) at 4°C overnight. After that, cells were incubated with goat anti-rabbit IgG (Abcam; 1:5,000) at 37°C for 1 h. Then, the nuclei were stained with DAPI (Beyotime, Shanghai, China) for 5 min. Finally, cells were observed under a fluorescence microscope (Olympus CX23, Tokyo, Japan).

### 2.10 Transwell assay

Transwell plates (24-well, Corning, New York, NY, USA) were used for cell invasion and migration detection. For the cell migration assay, 2 × 10^5^ A549 cells were seeded into the upper chambers of the 24-well plates in 200 µL of serum-free RPMI 1640 medium supplemented with 0.2% bovine serum albumin. The lower chambers contained RPMI 1640 medium supplemented with 1% FBS. After 24 h of incubation at 37°C, the nonmigrating cells were gently removed from the upper side of each chamber with a cotton swab, while the cells that had migrated were fixed with 95% alcohol for 10 min and stained with 1% crystal violet (Sigma, Grand Island, NY, USA) for 5 min. Finally, cells were counted under an inverted light microscope (Olympus) at 400× magnification.

For the invasion assay, the upper chambers of the 24-well plates were pretreated with 50 µL of Matrigel (12.5 mg/L, BD Biosciences, Franklin Lake, NJ, USA). Then, A549 cells (1 × 10^6^ cells/mL) in FBS-free medium were seeded into the upper chambers. The lower chambers contained RPMI 1640 medium supplemented with 1% FBS. The cells were incubated at 37°C for 24 h, and cells that had attached to the underside of the membrane were fixed and stained with 1% crystal violet solution. Finally, the number of invading cells was counted under a microscope at 400× magnifications.

### 2.11 Statistical analysis

At least three independent experiments were performed in each group, and all data were expressed as the mean ± standard deviation. Differences were analyzed using one-way analysis of variance followed by Tukey’s test (more than two groups, Graphpad Prism7). *P* < 0.05 was considered to indicate a statistically significant difference.

## 3 Results

### 3.1 Knockdown of FGF12-AS2 significantly inhibited the proliferation of NSCLC cells

**Figure 1:**
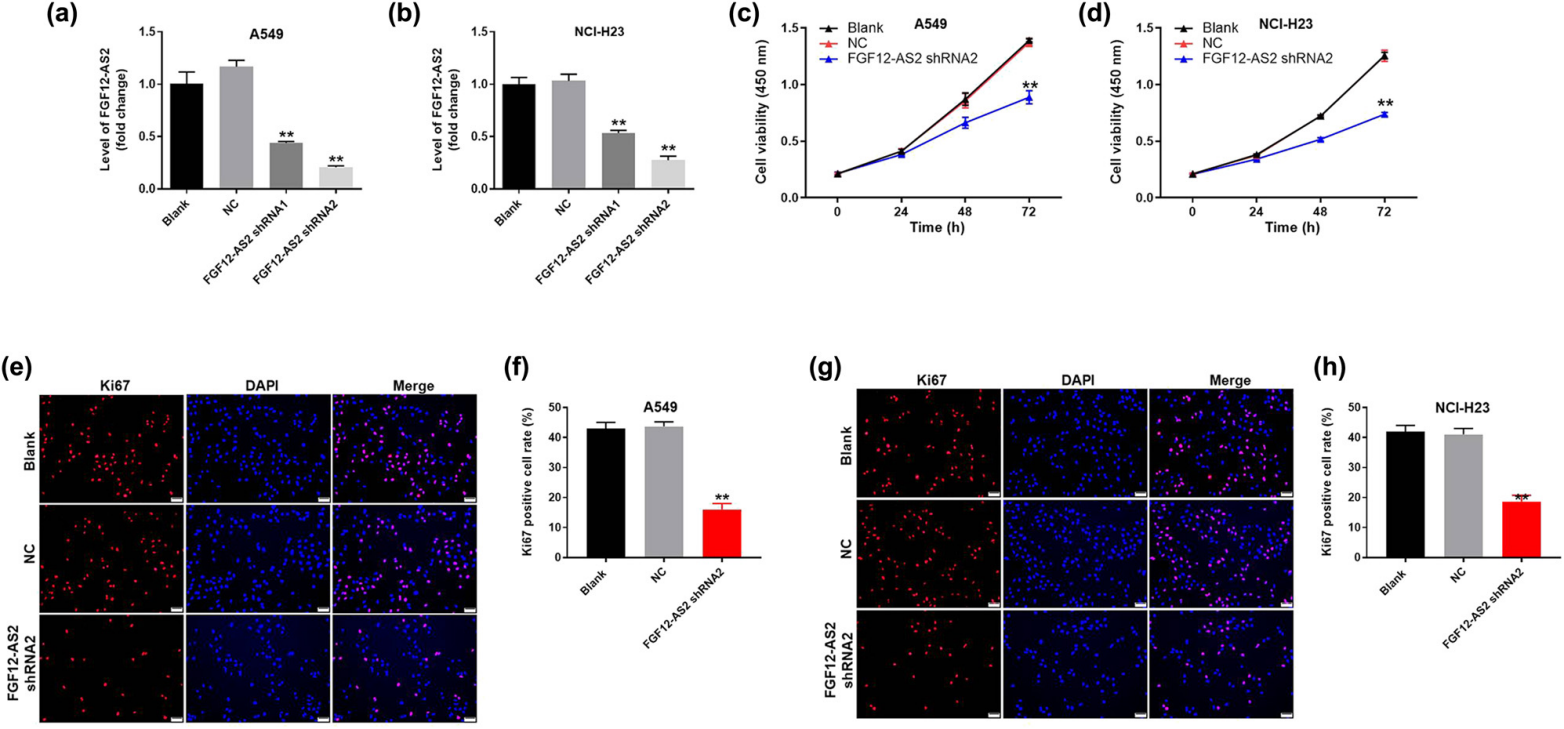
Knockdown of FGF12-AS2 significantly inhibited the proliferation of NSCLC cells. A549 or NCI-H23 cells were transfected with FGF12-AS2 shRNA1 or FGF12-AS2 shRNA2 for 24 h. Then, the expression of FGF12-AS2 in (a) A549 and (b) NCI-H23 cells was detected by q-PCR. (c and d) A549 or NCI-H23 cells were transfected with nothing, NC or FGF12-AS2 shRNA2 for 0, 24, 48, or 72 h. Then, the cell viability was detected by the CCK-8 assay. (e) The proliferation of A549 cells was tested by Ki-67 staining. Red immunofluorescence indicated Ki-67. Blue immunofluorescence indicated DAPI. (f) The positive rate of Ki-67 staining was calculated. (g) The proliferation of NCI-H23 cells was measured by Ki-67 staining. Red immunofluorescence indicated Ki-67. Blue immunofluorescence indicated DAPI. (h) The positive rate of Ki-67 staining was calculated. ^**^ *P* < 0.01 compared to control.

To verify the efficiency of cell transfection, q-PCR was performed. As shown in Figure 1a and b, the expression of FGF12-AS2 in NSCLC cells was significantly downregulated by knockdown of FGF12-AS2. Moreover, A549 and NCI-H23 cells were more sensitive to FGF12-AS2 shRNA2, compared with FGF12-AS2 shRNA1. Thus, FGF12-AS2 shRNA2 was selected to use in following experiments. Next, CCK-8 assay was used to measure the cell proliferation. The result demonstrated that cell viability of NSCLC was notably decreased in the presence of FGF12-AS2 knockdown (Figure 1c and d). Consistently, ki-67 positive rate of NSCLC cells was significantly downregulated by the silencing of FGF12-AS2 (Figure 1e–h). Since proliferation of A549 cells was more susceptible to lncRNA treatment, these cells were selected for use in following experiments. Altogether, these data suggested that the knockdown of FGF12-AS2 significantly inhibited the proliferation of NSCLC cells.

### 3.2 Silencing of FGF12-AS2 notably induced the apoptosis of NSCLC cells

**Figure 2:**
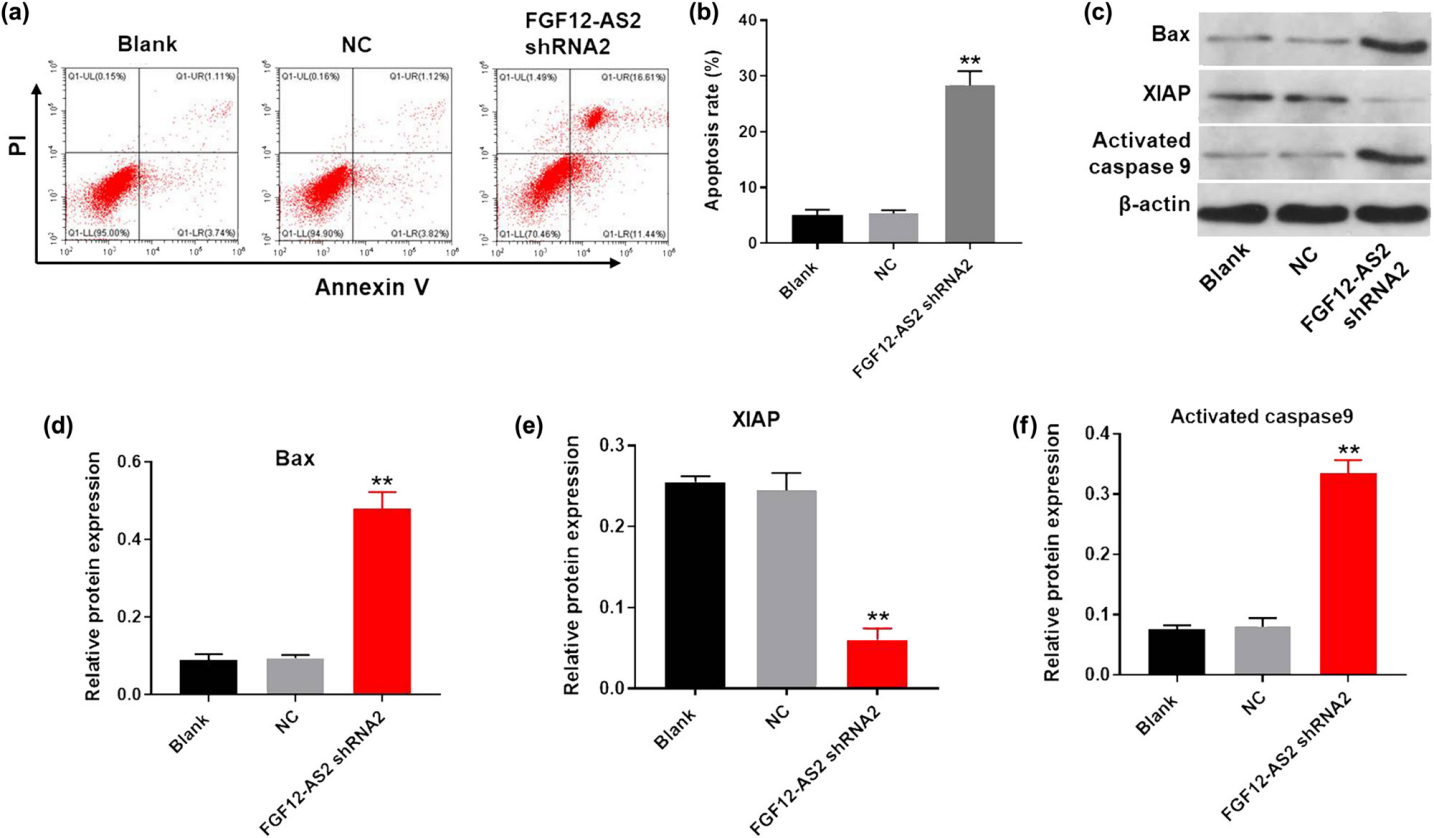
Silencing of FGF12-AS2 notably induced the apoptosis of NSCLC cells. (a and b) The rate of apoptotic cells was detected by FACS after double staining with Annexin V and PI. *X* axis: the level of Annexin-V FITC fluorescence; *Y* axis: the PI fluorescence. (c) The protein expression of Bax, XIAP, and Active caspase 9 in A549 cells was detected by western blot. (d) The relative expression of Bax was quantified normalizing to β-actin. (e) The relative expression of XIAP was quantified normalizing to β-actin. (f) The relative expression of active caspase 9 was quantified normalizing to β-actin. ^**^ *P* < 0.01 compared to control.

Next, to detect the cell apoptosis, flow cytometry was used. The results revealed that the downregulation of FGF12-AS2 greatly induced the apoptosis of A549 cells (Figure 2a and b). Moreover, the results of western blot detection revealed that the knockdown of FGF12-AS2 significantly upregulated the expression of pro-apoptotic proteins (Bax and Active caspase 9). In contrast, the expression of anti-apoptotic protein (XIAP) in A549 cells was obviously decreased in the presence of FGF12-AS2 silencing (Figure 2c–f). Taken together, silencing of FGF12-AS2 notably induced the apoptosis of NSCLC cells.

### 3.3 Downregulation of FGF12-AS2 notably inhibited the migration and invasion of NSCLC cells

**Figure 3:**
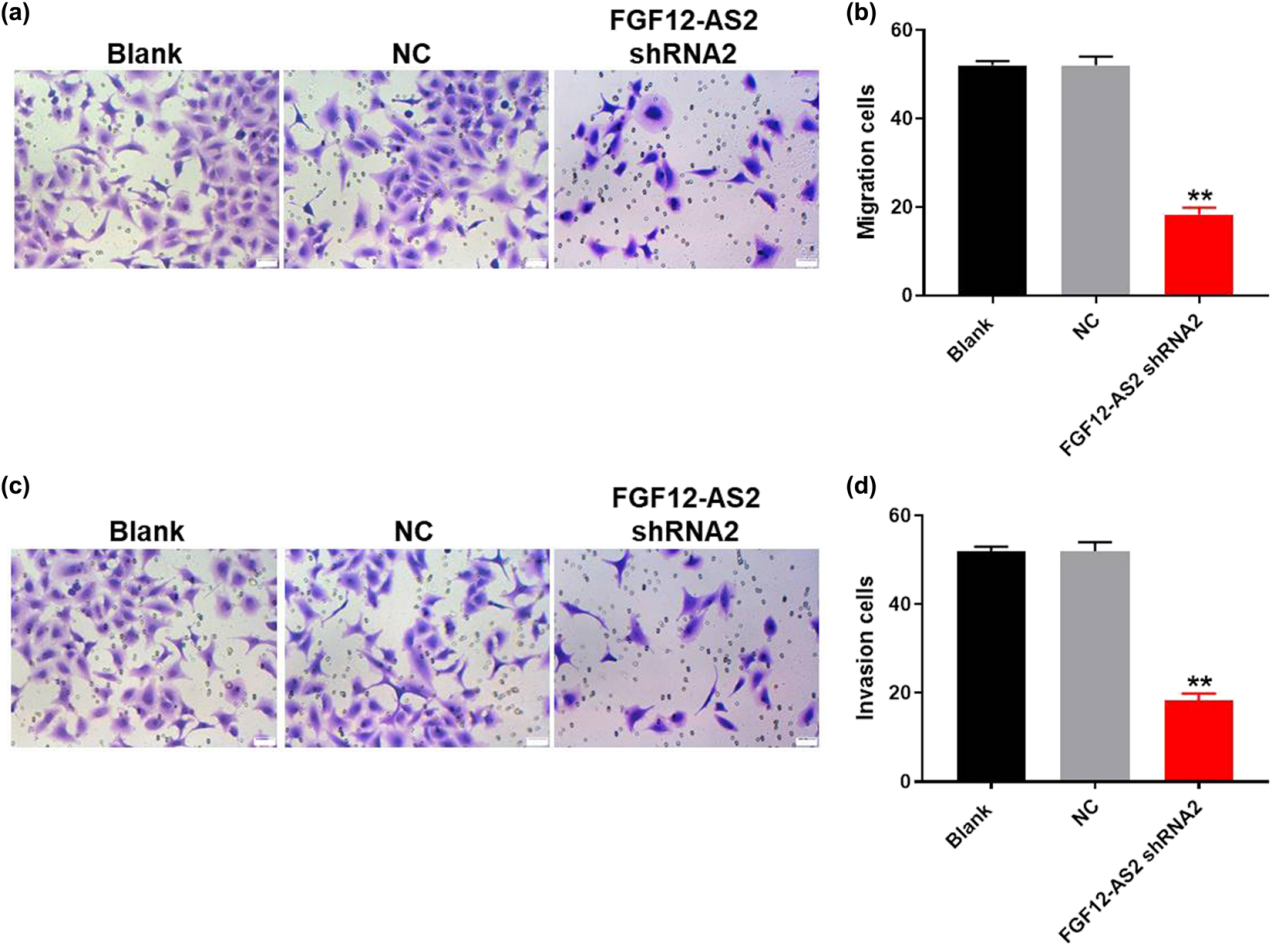
Downregulation of FGF12-AS2 notably inhibited the migration and invasion of NSCLC cells. (a and b) The migration of A549 cells was tested by transwell assay; ×400 magnification. (c and d) The invasion of A549 cells was tested using transwell invasion assay; ×400 magnification. ^**^ *P* < 0.01 compared to control.

To test cell migration and invasion, the transwell assay was performed. As shown in Figure 3a, the migration number of NSCLC cells was notably decreased by the knockdown of FGF12-AS2. Similarly, silencing of FGF12-AS2 significantly inhibited the cell invasion of NSCLC (Figure 3b). All these data confirmed that downregulation of FGF12-AS2 notably inhibited the migration and invasion of NSCLC cells.

### 3.4 LncRNA FGF12-AS2 could sponge miR-188-3p

**Figure 4:**
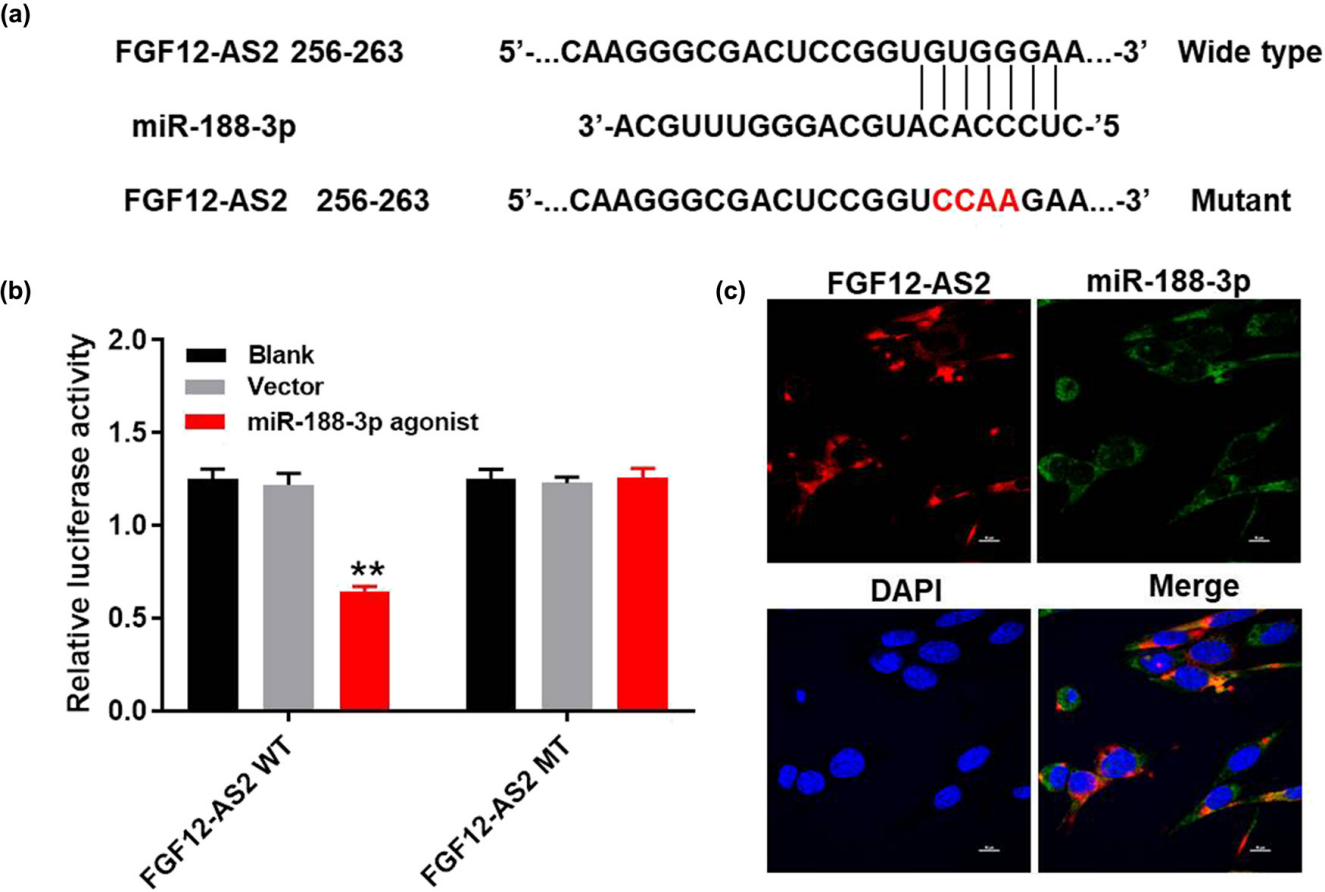
LncRNA FGF12-AS2 could sponge miR-188-3p. (a) Gene structure of lncRNA FGF12-AS2 at the position of 256–263 indicated the predicted target site of miR-188-3p in its 3′UTR, with a sequence of CCAA. (b) The luciferase activity was measured in A549 cells following co-transfecting with WT/MT FGF12-AS2 3′-UTR plasmid and miR-188-3p with the dual luciferase reporter assay. (c) The co-location of lncRNA FGF12-AS2 and miR-188-3p is detected by FISH detection. ^**^ *P* < 0.01 compared to control.

For the purpose of exploring the mechanism by which lncRNA FGF12-AS2 mediated the progression of NSCLC, miRDB (http://www.mirdb.org/) was performed. As shown in Figure 4a, lncRNA FGF12-AS2 had a putative miR-188-3p targeting site. In addition, the luciferase reporter assay was performed to determine whether miR-188-3p could directly interact with lncRNA FGF12-AS2 in A549 cells. The result indicated that co-transfection of the wild-type lncRNA FGF12-AS2 vector (WT-lncRNA FGF12-AS2) with miR-188-3p agonist significantly reduced luciferase activities compared with the mutant lncRNA FGF12-AS2 vector (MT-lncRNA FGF12-AS2) (Figure 4b). Besides, the result of FISH detection further verified that miR-188-3p was the downstream target of lncRNA FGF12-AS2 (Figure 4c). In summary, these data confirmed that lncRNA FGF12-AS2 could sponge miR-188-3p.

### 3.5 MiR-188-3p directly targeted non-SMC condensin II complex subunit G2 (NCAPG2)

**Figure 5:**
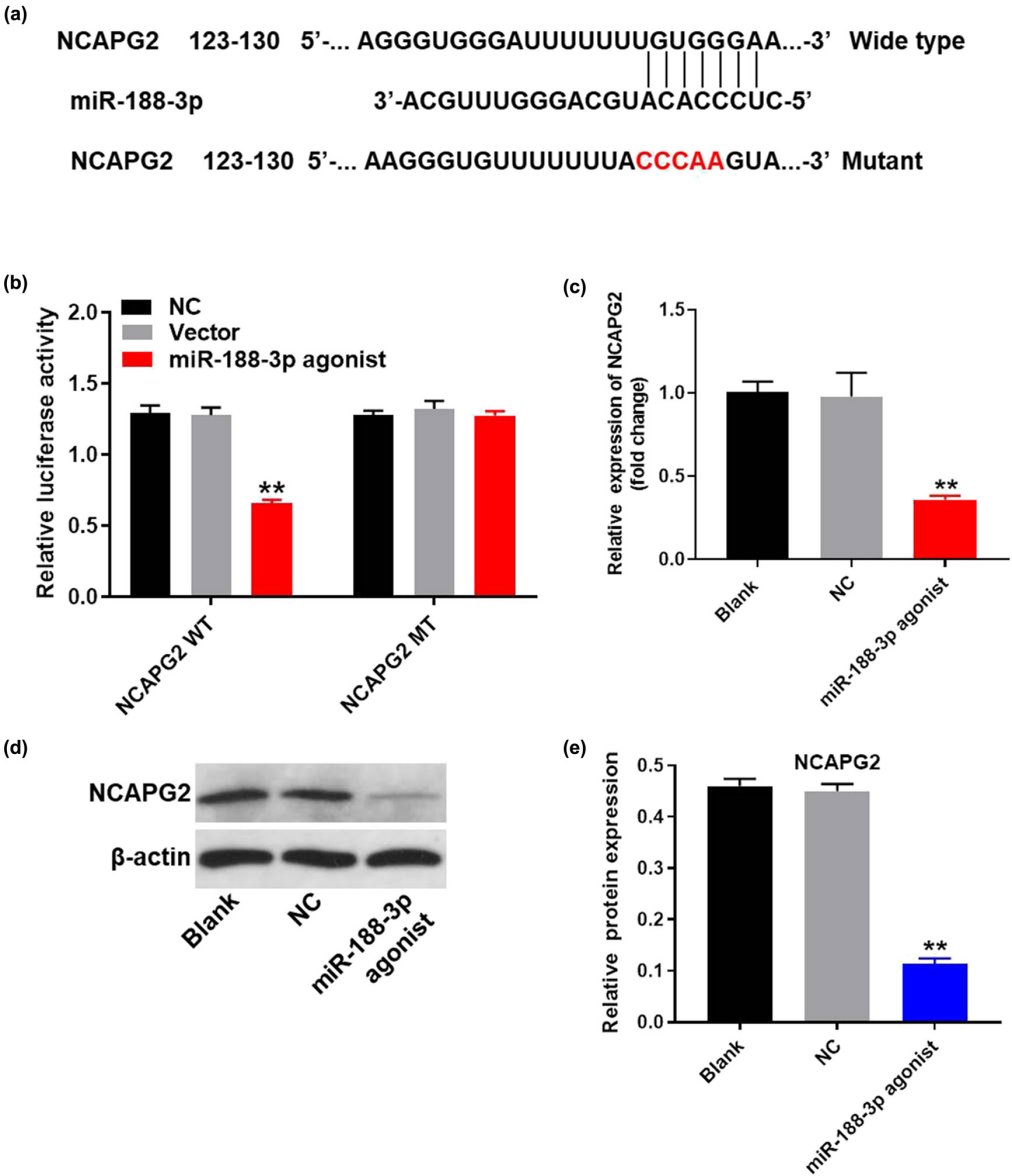
MiR-188-3p directly targeted NCAPG2. (a) Gene structure of NCAPG2 at the position of 123–130 indicated the predicted target site of miR-188-3p in its 3′UTR, with a sequence of CCCAA. (b) The luciferase activity was measured in A549 cells following co-transfecting with WT/MT NCAPG2 3′-UTR plasmid and miR-188-3p with the dual luciferase reporter assay. (c) A549 cells were transfected with miR-188-3p antagonist for 24 h. The expression of NCAPG2 in A549 cells was detected by q-PCR. (d) The protein expression of NCAPG2 in A549 cells was measured by western blot. (e) The relative expression of NCAPG2 was quantified normalizing to β-actin. ^**^ *P* < 0.01 compared to control.

To find the direct target of miR-188-3p, targetscan (http://www.targetscan.org/vert_71/), miRDB (http://www.mirdb.org/), and miRWalk (http://mirwalk.umm.uni-heidelberg.de/) were used. The result indicated that NCAPG2 might be a potential target of miR-188-3p (Figure 5a). In addition, the luciferase assay data indicated that the reduced luciferase activity was observed in A549 cells after transfection with WT-NCAPG2 and miR-188-3p agonist (Figure 5b). These data indicated that NCAPG2 were the direct target of miR-188-3p. Meanwhile, q-PCR and western blot were performed to verify this finding. As shown in Figure 5c–e, the expression of NCAPG2 in A549 cells was notably inhibited by miR-188-3p agonist. Altogether, miR-188-3p directly targeted NCAPG2.

### 3.6 LncRNA FGF12-AS2 enhanced the progression of NSCLC through promoting EMT process

**Figure 6:**
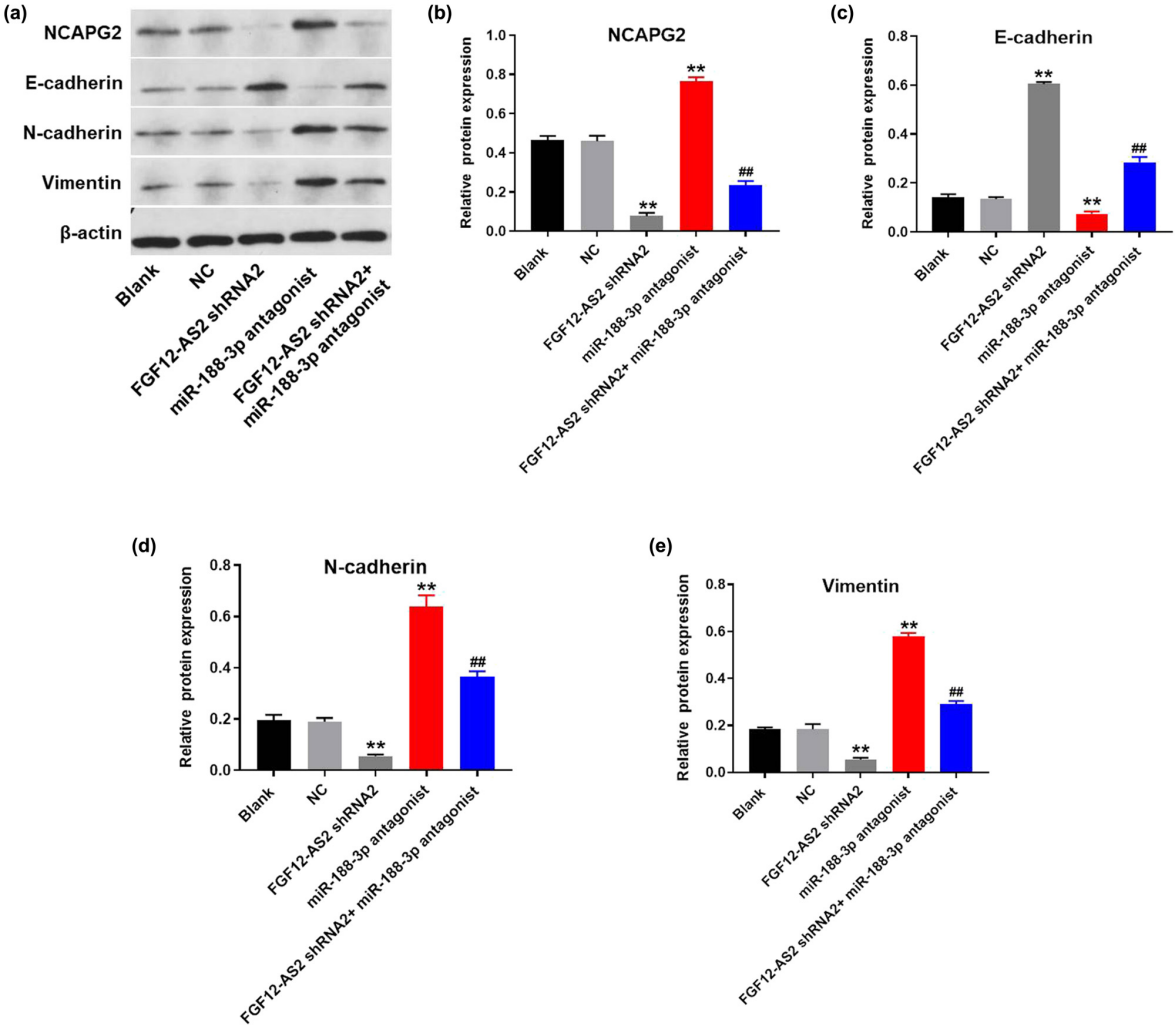
LncRNA FGF12-AS2 enhanced the progression of NSCLC through promoting EMT process. (a) The protein expressions of NCAPG2, E-cadherin, N-cadherin, and vimentin in A549 cells were detected by western blot. (b) The relative expression of NCAPG2 was quantified normalizing to β-actin. (c) The relative expression of E-cadherin was quantified normalizing to β-actin. (d) The relative expression of N-cadherin was quantified normalizing to β-actin. (e) The relative expression of vimentin was quantified normalizing to β-actin. ^**^ *P* < 0.01 compared to control.

To further explore the mechanism by which lncRNA FGF12-AS2 modulated the development of NSCLC, western blot was used. The data revealed that the expression of NCAPG2 in A549 cells was significantly inhibited in the presence of FGF12-AS2 silencing, which was partially rescued by downregulation of miR-188-3p (Figures 6a and b). In addition, the expression of E-cadherin in A549 cells was notably upregulated by the knockdown of FGF12-AS2, while miR-188-3p antagonist significantly reversed the effect of FGF12-AS2 on E-cadherin (Figure 6a and c). In contrast, silencing of FGF12-AS2 greatly inhibited the expression of vimentin and N-cadherin in A549 cells, while miR-188-3p antagonist exhibited the opposite effect. Moreover, FGF12-AS2-induced inhibition on these two proteins was significantly reversed by miR-188-3p antagonist (Figure 6a, d and e). Since E-cadherin, N-cadherin, and vimentin were major markers of EMT [16,17], these results suggested that the knockdown of FGF12-AS2 suppressed the progression of NSCLC through inhibiting the EMT process.

## 4 Discussion

It has been previously reported that lncRNAs participated in the modulation of gene expression and have the ability to encode proteins [18]. Moreover, lncRNAs are frequently expressed in many tumor tissues [19,20,21]. Some lncRNAs have important biological functions and can be considered as biomarkers for diagnosis of multiple diseases [22,23,24]. These details have supplied an interesting possibility that lncRNA, like circRNAs, may be involved in paracrine signaling or cell-to-cell crosstalk. In this study, we found that lncRNA FGF12-AS2 might act as a key regulator in the tumorigenesis of NSCLC. Next, our following experiments showed that FGF12-AS2 inhibition could inhibit the proliferation and induce the apoptosis of A549 cells. Many reports have confirmed that lncRNAs could regulate the progression of NSCLC [25,26,27]. A recent study indicated that FGF12-AS2 was upregulated in NSCLC [9]. Nevertheless, the role of FGF12-AS2 in the tumorigenesis of NSCLC remains to be further explored. Thus, our research supplemented the biological function of FGF12-AS2, suggesting that FGF12-AS2 could act as a promoter in tumorigenesis of NSCLC.

MiRNAs have been considered to play important roles in the progression of multiple diseases, including NSCLC [28,29]. In this study, we found that downregulation of miR-188-3p could partially reversed the antitumor effect of FGF12-AS2 knockdown. Meng et al. found that miR-188-3p could downregulate the cell proliferation and induce the apoptosis of hepatocellular carcinoma cells [30]. Yao et al. found that miR-188-3p was downregulated in NSCLC tissues [31]. In addition, our findings further confirmed the biological function of miR-188-3p, indicating that miR-188-3p could be a key regulator in the progression of NSCLC.

It has previously been reported that NCAPG2 could be participated in multiple malignant tumors [32,33]. Moreover, NCAPG2 has been found to regulate various biological functions, including cell proliferation, survival, and metastasis [34,35]. We have indicated that NCAPG2 was a direct target of miR-188-3p. It has been previously reported that miR-188-3p hepatocellular carcinoma proliferation and metastasis directly target NCAPG2 [30]. Moreover, NCAPG2 has been proved to be upregulated in NSCLC [33]. Our study was consistent with these previous data, further confirming that miR-188-3p directly targeted NCAPG2. Moreover, our current research first found that NCAPG2 expression was corrected with the progression of NSCLC, indicating that NCAPG2 might act as a key promoter in the occurrence of NSCLC.

EMT already participated in the very first steps of cancer formation. For example, ATP6L promotes metastasis of colorectal cancer via activation of epithelial–mesenchymal transition [36]. It has been considered that E-cadherin, N-cadherin, and vimentin were three major regulators during the EMT process [37,38,39]. Based on these data, it could be concluded in our research that silencing of lncRNA FGF12-AS2 significantly inhibited the EMT process in NSCLC. A previous report has confirmed that the upregulation of the EMT process could contribute to metastasis of NSCLC cells [40]. This result was consistent with our research, suggesting that the EMT process could act as an important regulator during the progression of NSCLC. Since it has been reported that TGF-β signaling could be involved in the EMT process of malignant tumors [41], we will further investigate the effect of lncRNA FGF12-AS2 on TGF-β signaling.

In conclusion, downregulation of lncRNA FGF12-AS2 suppresses the tumorigenesis of NSCLC via regulation of miR-188-3p/NCAPG2 axis. Thus, FGF12-AS2 may serve as a new target for the treatment of NSCLC.
